# Examination of Gait Characteristics Related to Sarcopenia in Community‐Dwelling Older Adults: A Study Focusing on Plantar Pressure

**DOI:** 10.1002/jcsm.13634

**Published:** 2024-11-22

**Authors:** Daiki Yamagiwa, Keitaro Makino, Osamu Katayama, Ryo Yamaguchi, von Fingerhut Georg, Yukari Yamashiro, Motoki Sudo, Hiroyuki Shimada

**Affiliations:** ^1^ Department of Preventive Gerontology, Center for Gerontology and Social Science National Center for Geriatrics and Gerontology Obu Aichi Japan; ^2^ Graduate School of Medical Welfare Science, Medical Engineering Hiroshima International University Higashihiroshima Hiroshima Japan; ^3^ Japan Society for the Promotion of Science Tokyo Japan; ^4^ Tokyo Research Laboratories Kao Corporation Tokyo Japan

**Keywords:** gait, gait strategy, plantar pressure, sarcopenia, walkway

## Abstract

**Background:**

Sarcopenia is a condition characterized by a decrease in skeletal muscle mass and strength with age, which results in a lower gait speed. Decreased gait speed in older individuals with sarcopenia can lead to adverse events such as falls and mortality. It is a major health issue; several studies have investigated gait speed in sarcopenia. However, plantar pressure has not been sufficiently evaluated. Plantar pressure facilitates gait analysis, including gait speed, and plays an important role in preventing adverse events such as falls and mortality. Therefore, the current study aimed to validate gait characteristics, including plantar pressure in community‐dwelling older adults with sarcopenia.

**Methods:**

The current study included community‐dwelling Japanese adults aged ≥60 years who participated in health checkups between 2013 and 2018. Sarcopenia was diagnosed by measuring muscle mass and strength based on clinical definition (nonsarcopenia: *n* = 7662; probable sarcopenia: *n* = 1208; and sarcopenia: *n* = 477). Gait parameters (including gait speed, relative plantar pressure, cadence, stride length, step length, step width and foot angle) were measured at a comfortable speed using a computerized electronic walkway. Gait parameters between groups were compared via an analysis of covariance adjusted for age and BMI. In addition, post hoc analyses were performed with Bonferroni correction.

**Results:**

The sarcopenia and probable sarcopenia groups had a significantly lower gait speed than the nonsarcopenia group (*p* < 0.01). Further, the sarcopenia and probable sarcopenia groups had a significantly lower forefoot plantar pressure, stride length and cadence than the nonsarcopenia group (all *p* < 0.01). When comparing, the sarcopenia group had a greater medial plantar pressure, step length, and foot angle and a lower lateral plantar pressure, cadence, and step width than the probable sarcopenia group (all *p* < 0.01).

**Conclusion:**

The sarcopenia, probable sarcopenia and nonsarcopenia groups differed concerning gait characteristics, including plantar pressure. It is thought that exercise instruction that takes into account walking characteristics is important for probable sarcopenia and sarcopenia.

## 1 Introduction

Sarcopenia was defined by Rosenberg as a type of age‐related skeletal muscle loss [1]. Subsequently, the European Working Group on Sarcopenia in Older People 2 (EWGSOP2) defined sarcopenia as decreased skeletal muscle mass and strength [2]. Decreased skeletal muscle mass and strength are risk factors for reduced gait ability [3]. Previous studies have shown that older adults with sarcopenia have a significantly lower stride than healthy older adults [4]. Hence, stride is associated with an increased risk of developing disabilities caused by falls and functional impairment [5, 6]. Therefore, sarcopenia is considered a common and serious health issue in older adults.

Changes in gait parameters associated with age include decreased gait speed, reduced stride length, increased stride width, decreased cadence and prolonged double stance time. Based on previous research on the associations between muscle mass and strength and gait ability, muscle strength is positively correlated with gait speed [7, 8]. However, whether these changes are caused by the ageing process or a preventive mechanism for falls or both is unclear [9]. Therefore, to expand our knowledge on changes in gait parameters, it is important to assess not only gait speed and muscle strength but also gait characteristics such as stride length.

Gait performance is a strong predictor of fall risk [10] and mortality [11]. Therefore, gait assessment is essential for health promotion in older adults. Previous reports have shown that lower gait speeds are associated with a higher risk of falls [12], and gait speed is a useful screening parameter. By contrast, when providing exercise intervention to older adults at a high risk of falling, gait speed alone is not sufficient. Gait is multidimensional and cannot be characterized by one parameter alone [13]. Investigating the gait dynamics underlying gait speed decline will facilitate exercise intervention, aiming to prevent falls. Plantar pressure is an indicator that encompasses spatiotemporal gait variables, such as gait speed [14, 15], cadence [16] and step length [17], and morphological characteristics, such as height, weight and fat‐free mass [18, 19]. Further, previous study has suggested that plantar pressure does not change significantly with changes in gait speed and that plantar pressure may be able to quantify walking characteristics that cannot be taken into account by gait speed [20]. Souza et al. [21] evaluated plantar pressure during gait in older adults with sarcopenia diagnosed based on SARC‐F and grip strength. Results showed that the plantar pressure in the rearfoot was greater than that in the forefoot, indicating that plantar pressure can be used to comprehensively investigate the characteristics of sarcopenia, such as decreased skeletal muscle mass and gait speed. However, no study has yet compared plantar pressure during gait in elderly adults with sarcopenia, probable sarcopenia and nonsarcopenia, as classified by the more comprehensive EWGSOP2 criteria.

The current study compared gait parameters including plantar pressure, according to nonsarcopenia, probable sarcopenia and sarcopenia classified by EWGSOP2. We hypothesized that nonsarcopenia and sarcopenia can be associated with a higher forefoot and rearfoot plantar pressures because of reduced gait speed and stride length. This may lead to the acquisition of walking characteristics that reduce the risk of falls and disabilities in patients with sarcopenia.

## 2 Methods

### 2.1 Participants

The current study used a cross‐sectional cohort dataset from the National Center for Geriatrics and Gerontology‐Study of Geriatric Syndromes (NCGG‐SGS). The NCGG‐SGS is a large cohort study that identifies the risks of geriatric syndromes and effective methods for treating them [22]. This study recruited 11 344 community‐dwelling older adults from Obu, Nagoya and Tokai City. The exclusion criteria were as follows: (1) patients with a history of stroke (*n* = 709), Parkinson's disease (*n* = 30) and dementia (*n* = 32); (2) those with cognitive impairment (Mini Mental State Examination score ≤ 18, *n* = 758); (3) those with missing data (*n* = 135); and (4) those who could not undergo measurement of body composition (*n* = 183) and gait parameters (*n* = 150). The data of 9347 older adults (mean age: 74.3 ± 5.3 years, 55.9% women) were analysed.

The current study was performed in accordance with the Declaration of Helsinki, and the ethics committee of the institutional review board of a national centre approved this study (1440–5). Informed consent was obtained from all participants prior to their study participation. We disclosed information about the study using the opt‐out approach, and the data of the participants who declined to participate directly or via proxy were excluded.

### 2.2 Sarcopenia Assessment

Sarcopenia was assessed according to muscle mass and strength and physical performance based on the EWGSOP2 recommendations. Our participants were Japanese. Thus, the cutoff values recommended by the revised Asian Working Group for Sarcopenia criteria (low muscle strength defined as a handgrip strength of <28 kg in men and <18 kg in women; low muscle mass defined as a skeletal muscle mass index [SMI] of <7.0 in men and 5.7 in women) were adopted [23].

Skeletal muscle mass was examined using a multifrequency bioelectrical impedance analyser (MC‐980A; TANITA, Tokyo, Japan), which can assess body composition. During the measurements, the participants were instructed to extend their arms and legs to prevent touching any other body part. The bioelectrical impedance analysis instrument used six electrical frequencies (1, 5, 50, 250, 500, and 1000 kHz). Regarding segmental body composition measurements, the participants' appendicular skeletal muscle mass was calculated using the following formula of the NCGG: appendicular skeletal muscle mass (men) = (0.197 × height^2^/50 kHz resistance) + (0.179 × weight) 0.019, and appendicular skeletal muscle mass (women) = (0.221 × height^2^/50 kHz resistance) + (0.117 × weight) + 0.881 [24]. The skeletal muscle mass of the participants was converted into SMI by dividing muscle mass (kilogramme) by height (metres squared).

The handgrip strength was measured using a Smedley‐type handheld dynamometer (Takei Ltd., Niigata, Japan). The participants underwent isometric measurements by holding a handheld dynamometer. Thus, the proximal interphalangeal joint of the index finger was at a 90° angle, and the highest measurement in the two assessments based on the dominant hand was used for analysis. Subsequently, to assess gait speed, participants walked at a comfortable pace along a flat, straight 2.4‐m gait path. Each trial was initiated and terminated 1 m before and after the start and end markers to minimize the effects of acceleration and deceleration. The average gait speed in the five trials was included as the representative value for the participants in the analysis.

**FIGURE 1:**
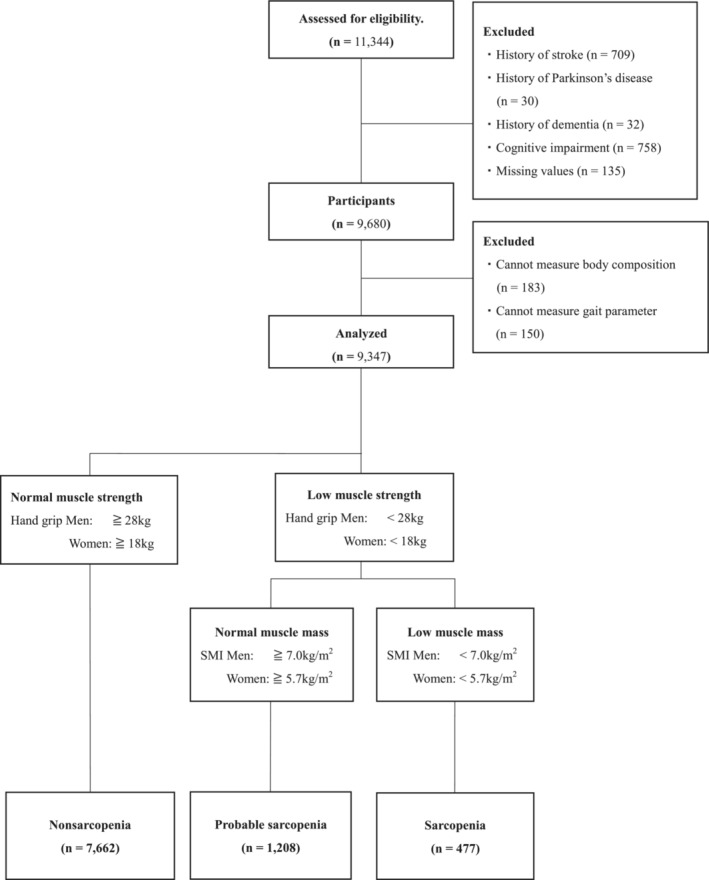
Participant flow diagram.

We classified the participants into three groups: the sarcopenia group, probable sarcopenia group and the nonsarcopenia group: sarcopenia, defined as presence of poor grip strength and low SMI; probable sarcopenia, defined as presence of poor grip strength alone; and nonsarcopenia, absence of the diagnostic criteria for either of the categories (Figure 1).

### 2.3 Gait Parameters Assessment

The gait parameters were measured using the Walkway device (Walkway MW‐1000; Anima Co., Tokyo, Japan). This device calculates temporal and spatial gait parameters from the distribution of plantar pressure and comprises a sheet size with a length of 2400 mm, width of 800 mm, thickness of 5 mm, 10‐mm sensor spatial resolution, and 14 400 measurement points. The participants were instructed to walk a total distance of 6.4 m (the gait section), which comprised a 2‐m acceleration section, 2.4 m of the Walkway device sheet (the measurement section), and 2 m of deceleration. The data were measured at a sampling rate of 100 Hz. The participants were instructed to walk barefoot at a regular speed, and two measurements at a normal gait speed were performed. The average of the two measurements was obtained. Then, the average of the left and right measurements was used as the representative value for each participant. The analysis was performed based on the following calculated factors: plantar pressure, cadence, stride length, step length, step width and foot angle (angle between the toes and the direction of travel). Plantar pressure was expressed in two parts: rearfoot/forefoot and medial foot/lateral foot. The rearfoot/forefoot was separated by an equivalent line from the lower end of the heel to the tip of the toe. The medial/lateral foot was delimited by a line connecting the lower end of the heel to the tip of the toe. These plantar pressures were normalized to the total plantar pressure during the stance phase [25]. Stride length, step length and step distance were normalized by height. For all gait parameters, the left and right average values were used as representative values.

### 2.4 Statistical Analysis

The characteristics of the participants among the three groups were examined using one‐way analysis of variance for continuous variables and the χ^2^ test for discrete variables. The gait parameters of the three groups were compared using analysis of covariance (ANCOVA) adjusted for age and body mass index (BMI). In addition, the effect sizes (ESs) were determined using eta squared from the ANCOVA power. ESs were small (0.01), medium (0.06) and large (0.14) [26]. Post hoc analyses were performed with Bonferroni comparisons. All analyses were performed using the Statistical Package for the Social Sciences software version 28 (IBM Inc., Tokyo, Japan). The level of statistical significance was set at *p* < 0.05.

## 3 Results

Of the participants enrolled in the NCGG‐SGS, 477 older adults presented with sarcopenia (5.1%), 1208 with probable sarcopenia (13.0%) and 7662 with nonsarcopenia (81.9%). There were significant differences in terms of age, height, weight, BMI, SMI, grip strength and gait speed (*p* < 0.001) (Table 1). The sarcopenia group had a higher proportion of male patients than the probable sarcopenia and nonsarcopenia groups (*p* < 0.001). In addition, the sarcopenia group had a significantly lower body weight, BMI and SMI than the probable sarcopenia and nonsarcopenia groups (*p* < 0.01). Among the three groups, the probable sarcopenia group had the highest proportion of women (*p* < 0.001). Furthermore, the probable sarcopenia group had the lowest height and grip strength among the three groups. However, the probable sarcopenia group had a significantly higher BMI and SMI than the sarcopenia and nonsarcopenia groups (*p* < 0.001). The nonsarcopenia group had a significantly higher grip strength and gait speed than the sarcopenia and probable sarcopenia groups (*p* < 0.001).

**TABLE 1:** Physical characteristics.

Characteristic	Nonsarcopenia (*n* = 7662)	Probable sarcopenia (*n* = 1208)	Sarcopenia (*n* = 477)		
				*F* value	*p*
Age (y)	73.7 ± 5.0	77.4 ± 5.5^N^	77.4 ± 5.9^N^	376.13	<0.001
Sex, male (%)	3383 (44.2)	474 (39.2)	264 (55.3)		<0.001
Height (cm)	157.4 ± 8.5	152.7 ± 8.6^N^	155.5 ± 8.6^N,P^	161.48	<0.001
Weight (kg)	57.7 ± 10.0	55.6 ± 9.3^N^	46.9 ± 9.3^N,P^	282.76	<0.001
BMI (kg/m^2^)	23.2 ± 3.0	23.8 ± 2.9^N^	19.3 ± 1.9^N,P^	434.44	<0.001
SMI (kg/m^2^)	6.3 ± 0.9	6.6 ± 0.9^N^	5.9 ± 0.6^N,P^	248.89	<0.001
Grip strength (kg)	28.3 ± 7.4	19.1 ± 5.0^N^	20.4 ± 5.1^N,P^	1097.37	<0.001
Gait speeds (m/s)	1.2 ± 0.2	1.0 ± 0.2^N^	1.0 ± 0.2^N^	292.41	<0.001

*Note*: Values are expressed as the mean ± standard deviation. N = difference versus nonsarcopenia; P = difference versus probable sarcopenia (*p* < 0.05). The *p* values of the post hoc comparisons are Bonferroni corrected.

Abbreviations: BMI, body mass index; SMI, skeletal muscle mass index.

**FIGURE 2:**
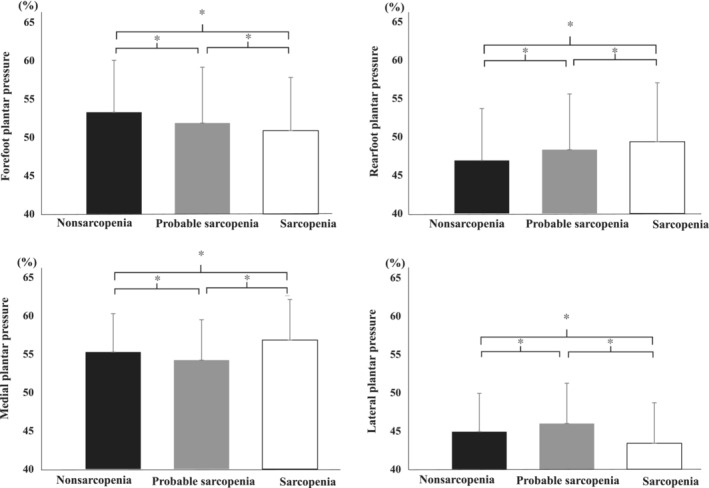
Comparison of plantar pressures in each group. Statistical analysis used one‐way analysis of variance. Black: nonsarcopenia. Grey: probable sarcopenia. White: sarcopenia. *p < 0.01. Lines indicate standard deviation.

Figure 2 and Table 2 show the results of the ANCOVA of gait parameters in the three groups adjusted for age and BMI. There was a significant difference in plantar pressure between the three groups. The nonsarcopenia group had a significantly higher rearfoot/forefoot plantar pressure than the sarcopenia and probable sarcopenia groups (*p* < 0.01). The sarcopenia group had a significantly greater plantar pressure in the medial foot/lateral foot than the nonsarcopenia and probable sarcopenia groups (*p* < 0.01). The probable sarcopenia group had the highest medial foot/lateral foot plantar pressure (*p* < 0.01).

**TABLE 2:** Gait parameters.

Characteristic	Nonsarcopenia (*n* = 7662)	Probable sarcopenia (*n* = 1208)	Sarcopenia (*n* = 477)	ANCOVA		
				*F* value	*p*	Effect size (η^2^)
Rearfoot plantar pressure (%)	46.9 ± 6.8	48.3 ± 7.3^N^	49.4 ± 7.7^N,P^	47.36	<0.001	0.018
Forefoot plantar pressure (%)	53.3 ± 6.9	51.9 ± 7.3^N^	50.9 ± 7.7^N,P^	45.83	<0.001	0.018
Medial plantar pressure (%)	55.3 ± 5.0	54.2 ± 5.3^N^	56.8 ± 5.3^N,P^	51.13	<0.001	0.008
Lateral plantar pressure (%)	44.9 ± 5.0	46.0 ± 5.3^N^	43.4 ± 5.3^N,P^	49.51	<0.001	0.008
Stride length (% Height correction)	78.8 ± 9.2	72.9 ± 10.6^N^	74.4 ± 10.7^N,P^	65.82	<0.001	0.021
Step length (% Height correction)	39.4 ± 4.6	36.4 ± 5.4^N^	37.2 ± 5.4^N,P^	66.49	<0.001	0.021
Step width (% Height correction)	5.0 ± 1.7	5.5 ± 1.9^N^	5.2 ± 1.8^P^	48.93	<0.001	0.007
Foot angle (°)	5.0 ± 5.7	5.2 ± 6.1^N^	6.2 ± 6.2 ^N,P^	8.47	<0.001	0.013
Cadence (steps/min)	137.4 ± 22.6	132.3 ± 22.0^N^	127.6 ± 22.8^N,P^	64.06	<0.001	0.007

*Note*: Values are expressed as the mean ± standard deviation. The symbols N and P represent a significant between‐group difference adjusted with age and BMI. N = difference versus nonsarcopenia; P = difference versus probable sarcopenia (*p* < 0.05). The *p* values of the post hoc comparisons are Bonferroni corrected.

Abbreviation: ANCOVA, analysis of covariance.

**FIGURE 3:**
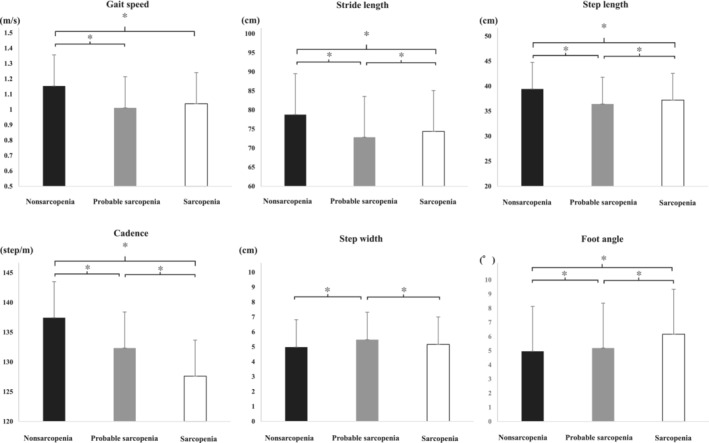
Comparison of gait parameters in each group. Statistical analysis used covariants adjusted for age and sex. Black: nonsarcopenia. Grey: probable sarcopenia. White: sarcopenia. *p < 0.01. Lines indicate standard deviation.

The three groups significantly differed in terms of the gait parameters other than plantar pressure (Figure 3 and Table 2). The nonsarcopenia group had a significantly greater stride length, step length and cadence than the sarcopenia and probable sarcopenia groups (*p* < 0.01). In addition, based on the post hoc test, the probable sarcopenia group had a significantly greater cadence and step width than the sarcopenia group (*p* < 0.01). However, the sarcopenia group had a significantly higher stride length, step length and foot angle than the nonsarcopenia and probable sarcopenia groups (*p* < 0.01).

## 4 Discussion

This study aimed to investigate plantar pressure during gait and gait characteristics in community‐dwelling older people with nonsarcopenia, probable sarcopenia and sarcopenia based on the EWGSOP2. Patients with sarcopenia and probable sarcopenia had a significantly lower forefoot plantar pressure, stride length, step length, cadence and gait speed than those with nonsarcopenia. Furthermore, the probable sarcopenia group had a significantly greater lateral plantar pressure, cadence and step width than the sarcopenia group. Meanwhile, the sarcopenia group had a significantly greater medial plantar pressure, step length and foot angle than the probable sarcopenia group.

Plantar pressure is an index that comprehensively reflects physical conditions (such as fat‐free mass and body weight) and gait characteristics (such as gait speed). Reportedly, plantar pressure in the forefoot region is positively correlated with body weight and gait speed [27]. Herein, the nonsarcopenia group had a significantly greater forefoot plantar pressure than the probable sarcopenia and sarcopenia groups. Further, among the three groups, the nonsarcopenia group had the highest stride length, cadence and gait speed. A direct comparison could not be performed because stride length and step length were corrected for height; the stride length and step length of patients with nonsarcopenia and sarcopenia in this study were slightly lower than those reported by Fan et al. [4]. Meanwhile, the cadence in this study was larger than the previous study [4]. These results may be influenced by older age in this study. Further, based on Mori et al.'s [28] study, the gait characteristics of probable sarcopenia in this study were similar to those of pre‐presarcopenia. Gait speed is an effective and reliable indicator of physical function and health in older adults [29] and is defined by stride length and cadence. Hence, a high forefoot plantar pressure in nonsarcopenia comprehensively reflects conditions such as body weight and gait speed, which is similar to previous studies [20, 30].

If gait speed reduces with age in older people, stride length and cadence decrease, and step width increases. Furthermore, decreased skeletal muscle mass and strength affect these gait characteristics [31]. Similarly, probable sarcopenia and sarcopenia are characterized by decreased gait speed. However, they differ in terms of plantar pressure and gait characteristics. Probable sarcopenia was associated with a greater lateral plantar pressure, cadence and step width compared with sarcopenia. By contrast, patients with sarcopenia had a greater medial plantar pressure, stride length and step length. If gait speed decreases, widening the step width reduces the risk of falling [32]. Elevating the step width increases the base of support and decreases the centre of mass variation. If the step width is large, a medial component of the ground reaction force should be generated to stabilize the centre of gravity. Therefore, the lateral plantar pressure inevitably increases, providing a gait characteristic that offsets the instability of gait that accompanies a decrease in gait speed. Therefore, by quantifying the gait dynamics associated with gait speed decline, the need for exercise instruction to improve gait function in patients with probable sarcopenia can be possibly identified.

By contrast, patients with sarcopenia had a greater medial plantar pressure, stride length and step length. Predominantly, older people with decreased gait speed adopt strategies that increase gait stability by decreasing step length. Considering that patients with sarcopenia have a lower skeletal muscle mass than those with probable sarcopenia, they could be desirable to show a lower stride and step length [33]. However, this study had contrasting results. Because of high medial plantar pressures, selecting an appropriate gait strategy because of poor foot function caused by foot pressure is challenging. In previous studies, high medial plantar pressures were associated with flat feet [20, 21, 22, 23, 24, 25, 26, 27, 28, 29, 30, 31, 32, 33, 34], which is a characteristic of older adults with decreased foot function. Therefore, patients with sarcopenia require exercise instruction to achieve gait stability, which is the top priority.

This study had several methodological limitations. First, it used a cross‐sectional design. Therefore, causal relationships between sarcopenia and gait parameters could not be identified. Therefore, future longitudinal research must be conducted to determine whether plantar pressure during gait is related to changes in gait in patients with sarcopenia. Second, the data on plantar pressure used in this study were collected from two periods: forefoot/rearfoot and medial/lateral. Therefore, it was challenging to investigate plantar pressure in a localized area such as the midfoot area. However, forefoot plantar pressure and gait velocity, as well as lateral plantar pressure and stride length, exhibit similar group characteristics. We believe that this study can provide important insights on plantar pressure in relation to the gait characteristics of older adults with sarcopenia.

## 5 Conclusion

The sarcopenia, probable sarcopenia and nonsarcopenia groups differed in terms of gait characteristics including plantar pressure. In particular, the probable sarcopenia and sarcopenia groups who presented with decreased gait speed had various plantar pressures and gait characteristics. Therefore, these findings may lead to individualized exercise guidance and intervention to prevent falls and disability, whether it is gait stability acquisition or exercise intervention to improve gait function, by quantifying the gait dynamics underlying the reduction in gait speed.

## Author Contributions

All authors reviewed the manuscript and made substantial contributions to the study's conceptualization, design, data acquisition, analysis, writing and interpretation and providing critical revisions for its intellectual content.

## Ethical Statement

We comply with the ethical guidelines for authorship and publishing in the Journal of Cachexia, Sarcopenia and Muscle.

## Conflicts of Interest

The authors declare no conflicts of interest.
